# Surgical Treatment of Radial Nerve Injuries Associated With Humeral Shaft Fracture—A Single Center Experience

**DOI:** 10.3389/fsurg.2021.774411

**Published:** 2021-12-16

**Authors:** Lukas Rasulić, Slavko Djurašković, Novak Lakićević, Milan Lepić, Andrija Savić, Jovan Grujić, Aleksa Mićić, Stefan Radojević, Vladimir Puzović, Miloš Maletić, Stefan Mandić-Rajčević

**Affiliations:** ^1^Faculty of Medicine, University of Belgrade, Belgrade, Serbia; ^2^Department of Peripheral Nerve Surgery, Functional Neurosurgery and Pain Management Surgery Clinic for Neurosurgery, University Clinical Center of Serbia, Belgrade, Serbia; ^3^Clinic for Neurosurgery, Clinical Center of Montenegro, Podgorica, Montenegro; ^4^Clinic for Neurosurgery, Military Medical Academy, Belgrade, Serbia; ^5^College of Higher Vocational Studies “Sports Academy”, Belgrade, Serbia; ^6^School of Public Health and Health Management and Institute of Social Medicine, Faculty of Medicine, University of Belgrade, Belgrade, Serbia

**Keywords:** radial nerve injuries, humeral shaft fracture, surgical treatment, outcome, neurolysis, grafting

## Abstract

Radial nerve injuries are often associated with humeral shaft fractures. The results of treatment of these injuries, by contemporary surgical approaches, remain diverse. In this paper we presented the outcomes and analyzed the patient, clinical, and surgical procedure related characteristics and factors that may influence the outcome overall, in 77 patients treated at Clinic for Neurosurgery, Clinical Center of Serbia during a 20 years period. The nerve injuries were verified by US and EMNG. The majority of patients were treated by neurolysis or sural nerve grafting, while only few were treated by direct suture. The final recovery was evaluated by muscle strength assessment and classified using MRC. We analyzed extension of the wrist, extension of the fingers including the thumb, and abduction of the thumb. There was a significant statistical difference in MRC grade following the treatment. The total rate of useful functional recovery was achieved in 69 (89.61%) out of all studied patients, out of whom 20 (28.99%) achieved excellent recovery, 26 (37.68%) achieved good recovery and 23 (33.33%) achieved fair recovery. Only 8 (10.39%) out of all studied patients achieved poor recovery. The injured nerves, that were preserved in continuity, acquired by a low-energy trauma, and treated earlier than the 6 months were associated with better functional outcome following the surgery. In addition, there was a trend of better functional improvement with aging, keeping in mind that the old were subjected to lower energy trauma. The expectant management followed by surgery of radial nerve injury associated with humeral shaft fracture should be around 3 months, and the surgical nerve repair should not be performed later than the 6 months after injury. The energy of trauma may be a factor predicting patient's final recovery following the treatment.

## Introduction

The fractures of the humeral shaft make up about 1–3% of all skeletal fractures, and belong to the group of the most common bone injuries (1–4). The incidence increases with age and may be associated with significant in-patient mortality and health care utilization costs (2, 5–7). In addition, the patients remain unable to return to work for a long period even after the surgery (8, 9), which is a significant socioeconomic issue (10).

Due to the close topographic ties between nervous, bony and vascular tissues (1, 11), the injuries of peripheral nerves are often associated with these injuries (12–14), and radial nerve injuries occur in between 2 and 18% of cases with humeral shaft fracture (15–19). This high rate of combined injuries is probably due to their close anatomic relation in the spiral groove (sulcus nervi radialis - SNR) at the posterior side of the humeral shaft, as well as due to the rigidity of the radial nerve while piercing the lateral intermuscular septum after exiting the groove (20–22). Despite the fact that the fracture repair is usually successful (23, 24), the injury to the radial nerve can leave permanent functional disability of the hand (wrist drop) and sequentially the arm as a whole (20). This loss of hand function is found to be a horrifying experience for the majority of patients (25), and the fact that most of the patients contribute significantly to the household and the community further exacerbates their own and their families suffering (26–28) and presents a big socioeconomic issue (25, 29, 30).

The expert opinions on the timing and necessity of the surgery for associated radial nerve injuries are divided. Some studies suggest that these lesions have a high rate of satisfactory spontaneous recovery (15, 16, 31), but it may take more than a year for the most of the patients to return to work (1, 32–34). Early exploration is only indicated in open fractures (15, 35), while the primary nerve repair is only indicated if the nerve has a clean-cut margin, both of which are rare when the nerve is injured by the bone fragments (32).

Based on the contemporary surgical approaches, and a vast personal experience, a clear strategy was developed to treat these patients, and we treated 77 patients during the last 20 years. Beside the outcomes, we aimed to analyze the patient, clinical, and surgical procedure related characteristics and factors that may influence the outcome overall.

## Materials and Methods

### Patients

We retrospectively analyzed hospital records in the period from January 1st, 2001 until December 31st, 2020 and found 147 patients with isolated radial nerve lesion, out of whom 77 met below mentioned criteria.

### Inclusion Criteria

- Patients surgically treated during a 20 years period (January 1st, 2001–December 31st, 2020)
- Minimal follow up of 1 year
- Unilateral non-pathological humeral shaft fracture
- Unilateral radial nerve palsy due to humeral shaft fracture or as a consequence of orthopedic management of the fracture

### Exclusion Criteria

- Compressive neuropathy
- Radial nerve injury without associated humeral shaft fracture
- Patients with previous history of peripheral nerve sheath tumor (PNST), demyelinating disorders, or neuropathy due to vasculitis or diabetes mellitus that have acquired humerus fracture and were sent to our clinic for examination
- Patients treated by artificial nerve graft

Before meeting the patients, we made a detailed review of their medical records, and formed a database. All data were re-checked and supplemented in subsequent contacts with the patients.

### Clinical Features

Prior to the surgery, all patients underwent a physical and a complete diagnostic evaluation. Humeral shaft fractures were verified by radiography, while nerve lesions were verified by ultrasonography (US) and neurophysiology, usually the electromyoneurography (EMNG).

To enquire a potential link between patient's characteristics and nerve recovery following the surgery, we considered the age, gender, smoking habits and presence of associated diseases.

The energy of the initial trauma was determined according to the etiology of injury (36): a low-energy trauma (fall from the standing position) and a high-energy trauma (falls from height, traffic accidents, and crushing injuries) and it was previously identified as a prognostic factor that may affect the final recovery (37).

For analyzing how preoperative nerve status affected patient's final recovery, we took into account nature of nerve injury, level of the nerve failure, and continuity of the nerve. Due to insignificant sensory disturbances following radial nerve injury, level of the nerve failure was evaluated by muscle strength assessment and graded according to British Medical Research Council muscle strength scaling system (MRC) (38). We analyzed extension of the wrist, extension of the fingers including the thumb, and abduction of the thumb. The final preoperative result for every single patient was achieved by summarizing MRC scores for all muscles tested.

Humeral shaft fractures were classified based on the level of the fracture line on the shaft (33): D1 (surgical neck fracture), D2 (proximal metaphysis fracture), D3 (fracture of the joint of the proximal and middle third of the body), D4 (fracture of the middle third of the body), D5 (fracture of the junction of the middle and distal third of the body), and D6 (distal metaphysis fracture). In order to analyze how associated injuries affected patient's final recovery, we took into account humerus fracture type and presence of other associated injuries.

### Treatment

The decision-making process and surgical strategy were determined according to the several principles.

Early surgical exploration was indicated in cases with open injuries, or iatrogenic cases with evident (or US confirmed) laceration or compression which were treated as soon as possible. When clear cut margins were present the patient underwent immediate direct suture.

In cases of traumatic nerve palsies associated with closed fracture of the humeral shaft, and iatrogenic nerve palsies without evident cause, a late exploration was indicated.

Following the failure of conservative treatment, after 3 months of expectance for EMNG signs of recovery to appear, the patients were referred to our institution for surgical evaluation and treatment.

This process had not changed much during the last 20 years, and there were no significant variations in treatment of this group of patients.

### Outcome Assessment

The final recovery was evaluated by muscle strength assessment and classified using MRC. The same muscles, tested preoperatively, were tested postoperatively, and the results were compared (total MRC score for all muscle groups tested). The modified scale of Highet and Holmes (Table 1), was used to classify the recovery, and fair or better results were deemed satisfactory (1, 32, 39).

**Table 1:** Combined scale for evaluating final recovery in study patients.

Poor	M0, M1 and M2 for all muscle groups
Fair	M3 for extension of the wrist and fingers; M0, M1, and M2 for thumb abduction
Good	M4 and M5 for extension of the wrist and fingers; M3 for thumb abduction
Excellent	M4 and M5 for all muscle groups

*Extension of the wrist, extension of the fingers including the thumb, and abduction of the thumb were examined*.

In order to examine how treatment modality and timing affected patient's final recovery, we took into consideration surgical procedures performed as well as the time elapsed until the surgery.

### Statistical Analysis

All statistical procedures were performed using IBM SPSS v26.0. Parameters of interest were described using the methods of descriptive statistics: mean, median, range, absolute (N) and relative (%) frequencies. The normality of data was assessed using Shapiro-Wilk test. For analyzing the association between patient's groups and patient's final recovery we performed Fisher's exact and Chi-Square test. For comparing preoperative and postoperative measurements, we used Wilcoxon signed-rank test. The significance factor was set to be lower than 0.05. Due to low occurrence, the 2 patients treated by direct suture were excluded from the statistical analysis.

For easier statistical analysis of certain factors and characteristics, the patients were divided into the groups: age (0–25, 26–50, and 51–75 years old), nature of nerve injury (traumatic/iatrogenic), humerus fracture type (D3 and/or proximally/D4 and/or distally), treatment timing (before 6 months/after 6 months), final recovery (excellent or good/fair or poor).

## Results

**Figure 1:**
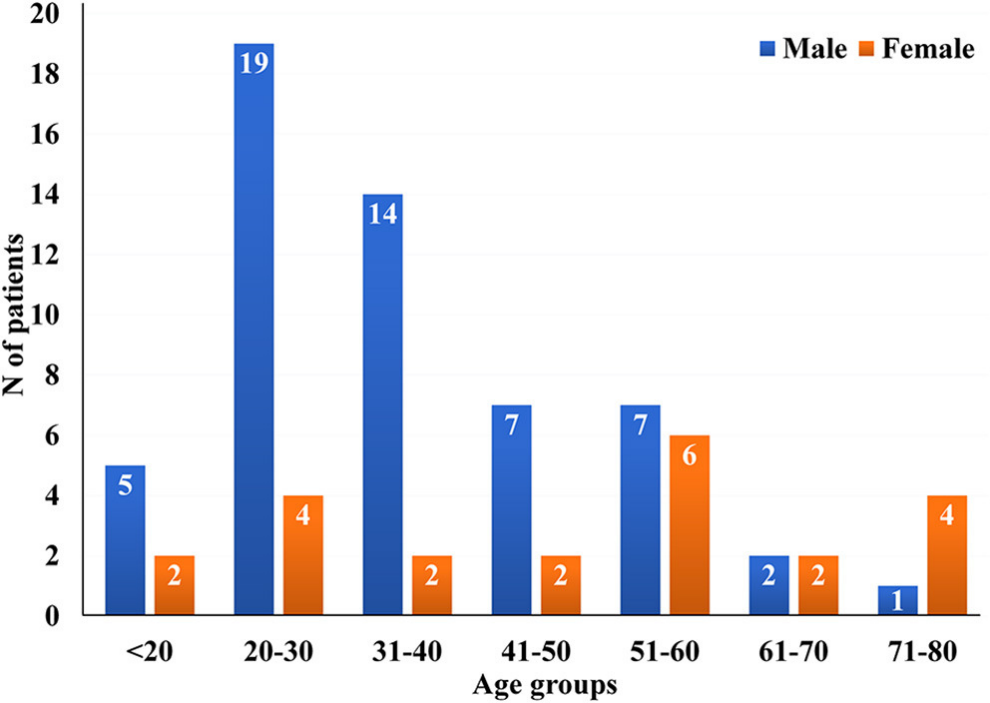
Age and sex distribution among included patients.

Total 55 (71.43%) male and 22 (28.57%) female patients were in the study group (Figure 1). The mean age was 39.39 ± 17.10, while their age ranged from 12 years old to the oldest patient of 75 years old. The mean and median age of male population were 35.38 ± 14.34 and 32.0 (12–32), while the mean and median age of female population were 49.41 ± 19.56 and 59.0 (18–75), respectively. More than a half of all studied patients−42 (54.55%) lived in urban places, while 35 (45.45%) of them lived in rural places.

Out of all studied patients, 31 (40.26%) were tobacco smokers. Twenty one patients (27.27%) had one associated disease, 4 (5.19%) had two, while 52 (67.53%) had none.

Concerning the energy of the initial trauma, 46 (59.74%) patients were subjected to injury by a high-energy trauma (male vs. female = 40:6), while 31 (40.26%) patients were subjected to injury by a low-energy trauma (male vs. female =15:16). The high-energy trauma was more common in the groups of patients aged 0–25 (70.0%) and 26–50 (74.29%), comparing to the group of patients aged 51–75 (27.27%). Furthermore, all patients with two or multiple associated injuries were subjected to injury by a high-energy trauma (Table 2).

**Table 2:** Distribution of associated injuries in patients with one, two and multiple associated injuries.

**Types of associated injuries**	***n*** **of associated injuries**			
	**1**	**2**	**Multiple**	**Total**
	**(*n* = 11)**	**(*n* = 9)**	**(*n* = 8)**	**(*n* = 14)**
**Long bone fractures**	4	8	13	25
Radius	4	4 (p1^*^, p2, p8, p9)	2 (p3, p7)	10
Ulna	/	4 (p1, p2, p8, p9)	4 (p2, p3, p7, p8)	8
Femur	/	/	4 (p1, p2, p6, p8)	4
Fibula	/	/	2 (p2, p8)	2
Clavicle	/	/	1 (p4)	1
**Axial skeletal fractures**	1	3	5	9
Cervical spine	/	/	3 (p1, p4, p6)	3
Thoracic spine	/	1 (p4)	/	1
Ribs	1	2 (p3, p4)	1 (p6)	4
Pelvis bones	/	/	1(p1)	1
**Joint luxation**	4	3	1	8
Elbow joint	2	1 (p3)	1 (p7)	4
Humeral joint	2	2 (p5, p7)	/	4
**Nerve injuries**	1	2	5	8
Median nerve	/	/	2(p1, p5)	2
Ulnar nerve	1	2 (p5, p7)	2(p2, p5)	5
Brachial plexus	/	/	1(p3)	1
**Muscles and tendons injuries**	1	/	1	2
Subscapular muscle	1	/	/	1
Deltoid muscle	/	/	1 (p4)	1
**Vascular injuries**	/	/	1	1
Brachial artery	/	/	1 (p5)	1
**Abdominal injuries**	/	2	/	2
Spleen	/	1(p6)	/	1
Mesentery	/	1(p6)	/	1

* *p1–p9 in the brackets represent injury that occurred in same patient*.

Regarding humeral shaft fracture type, 13 (16.89%) patients had fracture at the proximal third/middle third junction (D3), 42 (54.55%) patients had fracture at the middle third (D4), 20 (25.97%) patients had fracture at the middle third/distal third junction (D5), and only 2 (2.59%) patients had fracture at the distal third (D6) of the humeral shaft.

Primary nerve injury occurred in 45 (58.44%) patients, while secondary (iatrogenic) nerve injury occurred in 32 (41.56%) patients (Table 3). Out of all studied patients, 59 (76.62%) acquired complete nerve palsy (M0 for all muscle groups), while only 18 (23.38%) acquired incomplete nerve palsy (M1–M3 for all muscle groups).

**Table 3:** Distribution of the study patients in reference to etiology of nerve injury, nature of nerve injury, nerve continuity and level of nerve failure.

**Nature of nerve injury**		**Etiology of nerve injury**	**Nerve failure level**		**Nerve continuity**	
			**Complete**	**Incomplete**	**Preserved**	**Interrupted**
Primary injury	*n* = 45 (100%)	Traffic accident	11 (24.4)	5 (11.1)	12 (26.7)	4 (8.9)
		Fall	9 (20.0)	1 (2.2)	8 (17.8)	2 (4.4)
		Occupational accident	11 (24.4)	2 (4.4)	7 (15.5)	6 (13.3)
		Other	3 (6.7)	3 (6.7)	3 (6.7)	3 (6.7)
		Total	34 (75.6)	11 (24.4)	30 (66.7)	15 (33.3)
Secondary injury	*n* = 32 (100%)	Internal fixation	20 (62.5)	7 (21.9)	23 (71.9)	4 (12.5)
		Osteosynthetic material removal	5 (15.6)	/	/	5 (15.6)
		Total	25 (78.1)	7 (21.9)	23 (71.9)	9 (28.1)

Most of the patients had the nerve preserved in continuity, and all these patients were treated with neurolysis procedures, while the patients with completely interrupted continuity were subjected to the nerve repair. Out of 53 patients with the nerve preserved in continuity, 35 (66.04%) were treated by external neurolysis, 10 (18.87%) were treated by longitudinal epineurotomy, and 8 (15.09%) were treated by circumferential epineurectomy and interfascicular neurolysis. Two patients (8.33%) had direct nerve suture immediately during the initial exploration of the cut nerve. Other 22 patients (91.67%) with interrupted continuity underwent grafting. There were no complications related to the nerve surgery. Table 4 shows the distribution of the study patients in reference to the surgical procedure performed, nature of the nerve injury, level of the nerve failure, and time passed to the surgery.

**Table 4:** Distribution of the study patients in reference to the surgical procedure performed, nature of the nerve injury, level of the nerve failure and time to surgery.

**Surgical procedure**	**Nature of nerve injury (palsy)**		**Nerve failure level (palsy)**		**Time to treatment (months)**			
	**Primary *n* (%)**	**Secondary *n* (%)**	**Complete *n* (%)**	**Incomplete *n* (%)**	**0–3**	**3–6**	**6–9**	**>9**
Direct suture	2 (4.4)	/	2 (3.4)	/	2	/	/	/
Neurolysis	30 (66.7)	23 (71.9)	35 (59.3)	18 (100)	7	35	3	8
Grafting	13 (28.9)	9 (28.1)	22 (37.3)	/	/	12	8	2
Total	45 (100)	32 (100)	59 (100)	18 (100)	9	47	11	10

The signs of motor recovery were accomplished in all studied patients. There was a significant increase in MRC grade following surgical treatment (Z = −7.544, *p* < 0.01). The total rate of useful functional recovery was achieved in 69 (89.61%) out of all studied patients, out of whom 20 (28.99%) achieved excellent recovery, 26 (37.68%) achieved good recovery and 23 (33.33%) achieved fair recovery. Only 8 (10.39%) out of all studied patients achieved poor recovery.

**Figure 2:**
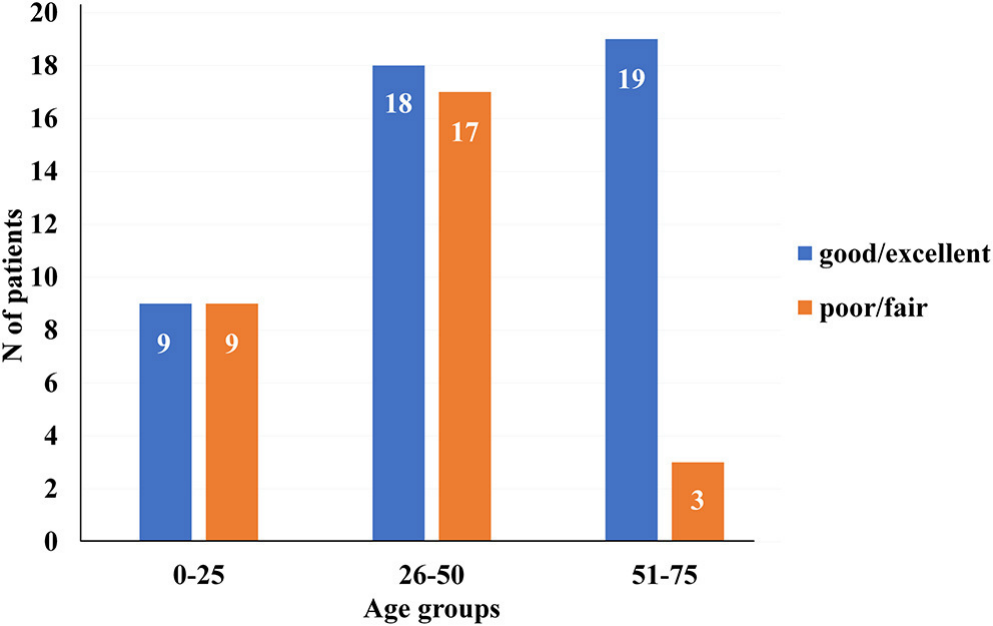
Distribution of the patients in reference to patient's age and patient's final recovery [*x*2 (2, *N* = 75) = 8.235, *p* = 0.016].

Regarding patient's characteristics such as gender (*p* = 0.192), smoking habits (*p* = 0.150), and presence of associated diseases (*p* = 0.065), there were no significant statistical differences in patients' final recovery. However, there was a significant difference with reference to patient's age (Figure 2). The excellent and good results were more common in the group of patients that were aged 51–75.

**Figure 3:**
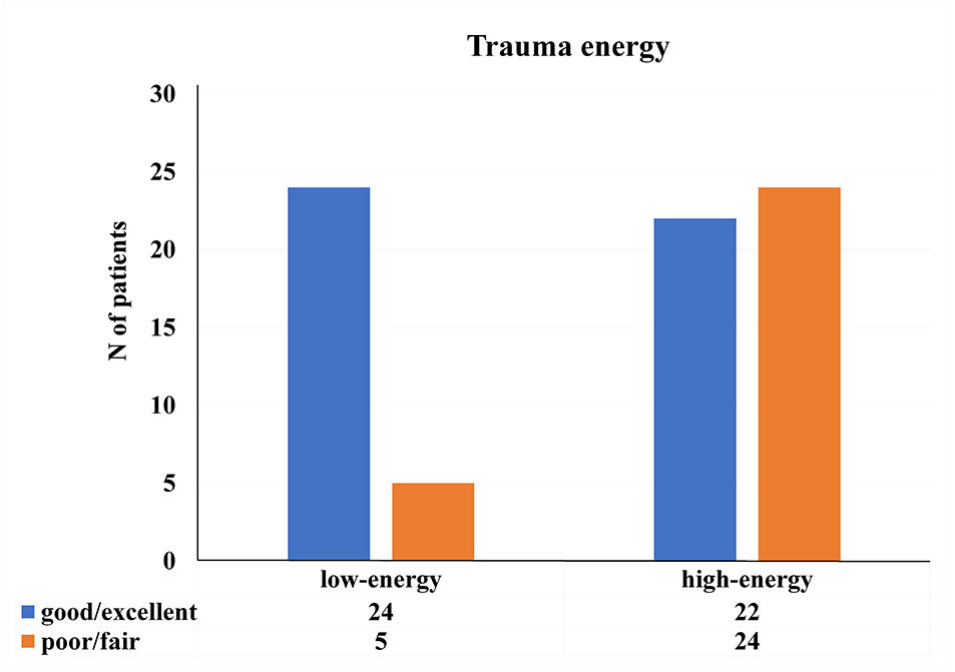
Distribution of the patients regarding energy of the initial trauma and patient's final recovery [*x*2 (1, *N* = 75) = 9.152, *p* < 0.01].

As for the concern of energy of the initial trauma, there was a significant statistical difference in patients' final recovery (Figure 3), while, there were no significant differences regarding humerus fracture type (*p* = 0.801) and presence of other associated injuries (*p* = 0.120). The majority of patients subjected to injury by a low-energy trauma−24 (82.76%) achieved excellent or good results.

**Figure 4:**
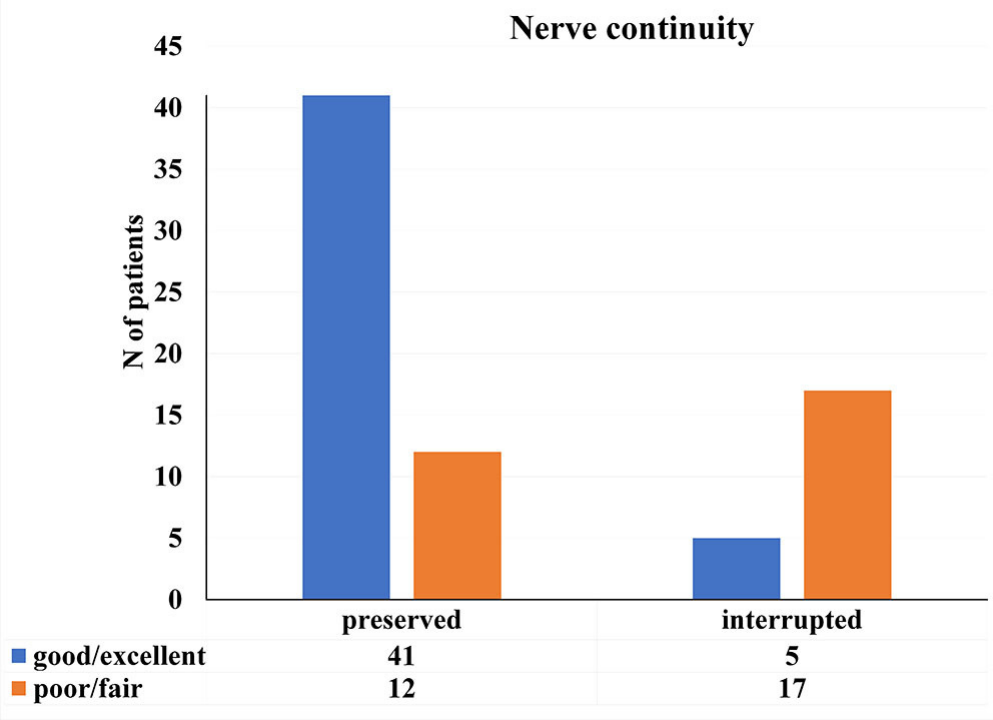
Distribution of the patients in reference to nerve continuity and patient's final recovery [*x*2 (1, *N* = 75) = 19.565, *p* < 0.01].

Regarding parameters such as nature of nerve injury (*p* = 0.764) and level of the nerve failure (*p* = 0.982), there were no significant differences in patient's final recovery following surgery. However, there was a significant difference with reference to continuity of the nerve (Figure 4). The majority of patients with the nerve preserved in continuity−41 (77.36%) achieved excellent or good results.

**Figure 5:**
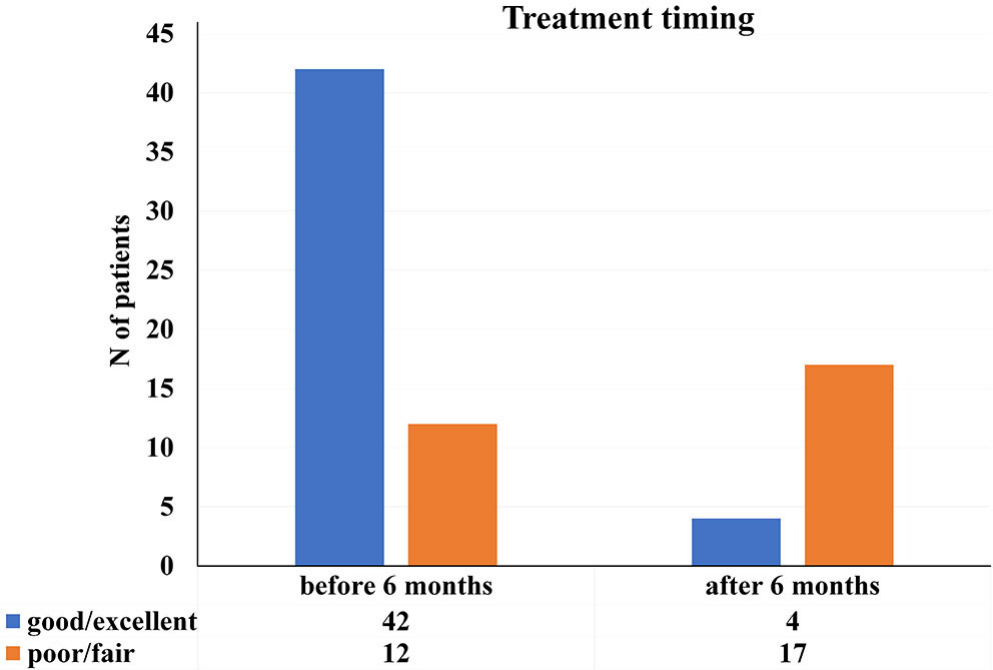
Distribution of the study patients according to patient's final recovery and timing of the treatment [*x*2 (*N* = 75), *p* < 0.01].

Regarding timing of the treatment, there was a significant difference in patients' final recovery between the groups treated earlier and groups treated later than the 6 months since the injury (Figure 5). The most of the patients treated earlier than the 6 months−42 (77.78%) achieved excellent or good results.

Distribution of the patients regarding age, treatment timing, nerve continuity and final recovery is presented in the Table 5.

**Table 5:** Distribution of the patients in reference to age, treatment timing, nerve continuity and final recovery.

***n*** **of patients** **=** **77**		**Poor/fair**				**Good/excellent**			
		**High-energy**		**low-energy**		**High-energy**		**Low-energy**	
		p.c.^*^	i.c.^**^	**p.c**.	**i.c**.	**p.c**.	**i.c**.	**p.c**.	**i.c**.
0–25	<6 months	/	2	/	2	5	2	2	/
	>6 months	1	4	2	/	/	/	/	/
26–50	<6 months	2	4	2	/	11	2	3	/
	>6 months	4	5	/	/	/	/	2	/
51–75	<6 months	1	/	/	1	4	/	12	1
	>6 months	/	1	/	/	/	/	2	/

* *p.c., preserved continuity*;

** *i.c., interrupted continuity*.

## Discussion

The vary fact that more than a half of radial nerve lesions treated at our clinic during the last 20 years were associated with humeral shaft fracture, indicates the importance of this particular entity.

Recently published studies (16, 18, 31, 34, 35, 37, 40), concerning the outcome in patients with radial nerve injury associated with humeral shaft fracture, have used different inclusion and exclusion criteria comparing to our study. Most of these studies included only conservatively treated patients (16, 18, 31), while some of them included only complete nerve failures (34, 35, 37), or only primary nerve injuries due to humerus fracture (18, 31). The studies that included both surgically and conservatively treated patients were mainly concentrated on determining the best indication for early nerve exploration and repair (34, 35, 40). Therefore, it was difficult to compare all these results with each other, as well as with the results of our study. However, we were able to compare the results of our study with the results of two other studies (1, 32), which have also included only surgically treated patients and have evaluated patient's final recovery using MRC muscle scale.

According to the published literature (41–44), the aging influences morphologic and functional features of the peripheral nerves, which may alter final regeneration and recovery of the nerves. However, according to our results, there was a trend of improved functional recovery with aging. Although apparently misleading, the older population is more cautious, and the trauma is usually a low-energy event (45, 46), therefore, the injury as well as eventual surgery is less extensive, and with better recovery potential. Contributing to this are the results of the study by Joseph et al. (47) which have revealed that age, as an independent factor, was not predictive of functional outcome after injury.

The most of our younger patients were subjected to injury by a high-energy trauma, which was associated with poorer final recovery. A poorer final recovery in patients subjected to injury by a high-energy trauma has also been shown in another study conducted over the same subject, and according to those authors (37) it may be caused by the extensive zone of tissue injury.

The quality of functional recovery was better in patients with the injured nerve preserved in continuity compared to the interrupted cases, which is in accordance with the results of previous studies (20, 48–50).

Regarding previous studies, concerning associated humeral shaft fractures and radial nerve injuries (1, 32, 35), in case of no indications for primary exploration, the expectant management of nerve injury followed by surgical treatment should last for 3–4 months, and the treatment should not be performed later than 5–6 months (51). We agree with these recommendations, and therefore, we treated most of our patients in the period between 3 and 6 months. We were not able to perform early exploration in all situations where it was indicated (1, 32, 35), because many of our patients lived in rural places, and it took more time for these patients to be referred to our institution, as local physicians were not always aware of recent indications for closed injuries. Regarding the 9 patients treated earlier than the 3 months since the injury, 2 of them had the nerve with clean-cut margins, which was an indication for primary nerve repair, 3 had the nerve compressed by a plate, and 4 others had an immediate radial nerve palsy following conservative treatment by other specialists, confirmed by clinical and EMNG findings. However, some of our patients were treated later than the 6 months since the injury, which may be also due to different place of patient's residence, as well as due to different extent of patient's injury and number of other associated injuries. The cause of eventual later management of nerve injuries in the patients who lived in rural places might be due to difficulties for general practitioners to diagnose peripheral nerve injuries, and therefore it takes more time for those patients to be referred to our institution. The cause of eventual later management of nerve injuries in all patients may be due to polytrauma and delayed deployment of these patients from the institutions responsible for the care of bone fractures (1, 52).

The results of our study regarding the total rate of useful functional recovery are comparable with the results of previous studies (1, 32) that have used modified Highet's scale in order to qualitative describe patient's final recovery. Despite the fact that rate of useful functional recovery in patients with the nerve preserved in continuity was similar, the rate of useful functional recovery in patients with the nerve interrupted in continuity was lower in our study comparing to the results of aforementioned studies (1, 32). The differences in these results may be due to different length of the nerve gap, as well as due to different energy of the initial trauma.

Considering that, in their study, neither of these authors presented energy of the trauma, we emphasize the importance of presenting it and considering it as a prognostic factor that may predict patients' final recovery following surgery.

## Conclusion

The expectant management followed by surgery of radial nerve injury associated with humeral shaft fracture should be around 3 months, and the surgical nerve repair should not be performed later than the 6 months after injury. The energy of trauma may be a factor predicting patient's final recovery following the treatment.

## Data Availability Statement

The original contributions presented in the study are included in the article/supplementary material, further inquiries can be directed to the corresponding author/s.

## Ethics Statement

The studies involving human participants were reviewed and approved by Ethics Committee of the Faculty of Medicine, university of Belgrade. The patients/participants provided their written informed consent to participate in this study.

## Author Contributions

All authors contributed to the preparation of this manuscript. The manuscript has been seen and approved by all authors.

## Conflict of Interest

The authors declare that the research was conducted in the absence of any commercial or financial relationships that could be construed as a potential conflict of interest.

## Publisher's Note

All claims expressed in this article are solely those of the authors and do not necessarily represent those of their affiliated organizations, or those of the publisher, the editors and the reviewers. Any product that may be evaluated in this article, or claim that may be made by its manufacturer, is not guaranteed or endorsed by the publisher.
